# Source localization and functional network analysis in emotion cognitive reappraisal with EEG-fMRI integration

**DOI:** 10.3389/fnhum.2022.960784

**Published:** 2022-08-12

**Authors:** Wenjie Li, Wei Zhang, Zhongyi Jiang, Tiantong Zhou, Shoukun Xu, Ling Zou

**Affiliations:** ^1^School of Microelectronics and Control Engineering, Changzhou University, Changzhou, China; ^2^School of Computer Science and Artificial Intelligence, Changzhou University, Changzhou, China; ^3^Key Laboratory of Brain Machine Collaborative Intelligence Foundation of Zhejiang Province, Hangzhou, China

**Keywords:** cognitive reappraisal, EEG-fMRI, source localization, functional network, phase lag index

## Abstract

**Background:**

The neural activity and functional networks of emotion-based cognitive reappraisal have been widely investigated using electroencephalography (EEG) and functional magnetic resonance imaging (fMRI). However, single-mode neuroimaging techniques are limited in exploring the regulation process with high temporal and spatial resolution.

**Objectives:**

We proposed a source localization method with multimodal integration of EEG and fMRI and tested it in the source-level functional network analysis of emotion cognitive reappraisal.

**Methods:**

EEG and fMRI data were simultaneously recorded when 15 subjects were performing the emotional cognitive reappraisal task. Fused priori weighted minimum norm estimation (FWMNE) with sliding windows was proposed to trace the dynamics of EEG source activities, and the phase lag index (PLI) was used to construct the functional brain network associated with the process of downregulating negative affect using the reappraisal strategy.

**Results:**

The functional networks were constructed with the measure of PLI, in which the important regions were indicated. In the gamma band source-level network analysis, the cuneus, the lateral orbitofrontal cortex, the superior parietal cortex, the postcentral gyrus, and the pars opercularis were identified as important regions in reappraisal with high betweenness centrality.

**Conclusion:**

The proposed multimodal integration method for source localization identified the key cortices involved in emotion regulation, and the network analysis demonstrated the important brain regions involved in the cognitive control of reappraisal. It shows promise in the utility in the clinical setting for affective disorders.

## Introduction

Emotion regulation is crucial to social functioning, mental health, and wellbeing. Cognitive reappraisal is an antecedent-focused emotion regulation strategy that aims to modulate emotional processes before the full emotional response occurs (Kim et al., 2021) by reinterpreting the meaning of an emotional event (McRae and Gross, 2020). Previous studies examined the time course of cognitive reappraisal and investigated the neural interaction in brain networks using single-mode neuroimaging techniques, such as electroencephalography (EEG) and functional magnetic resonance imaging (fMRI) (Langeslag and Surti, 2017; Steward et al., 2021). However, due to the limitations of single-mode neuroimaging in time and space resolution, some of the detailed neural characteristics involved in cognitive reappraisal remain unclear.

EEG estimates cortical activity with the temporal resolution of milliseconds, so the temporal evolution of neural activity during reappraisal is frequently investigated using EEG. The late positive potential (LPP) is one of the most important event-related potentials (ERP), which appears 300 ms after the stimulus onset with the central-parietal scalp distribution (Chen et al., 2020; Schindler and Bublatzky, 2020). With the development of brain functional networks and graph theory analysis, more attention was paid to the system interactions in the process of reappraisal. However, functional connectivity derived from EEG signals is limited by the volume conduction problem, and the channel locations cannot be seen as an approximation of a source's anatomical location. Besides, spurious connectivity can occur between sensors, because the EEG recordings on scalp electrodes are a mixture of signals from many source activities (Van de Steen et al., 2019). From this perspective, source-level functional connectivity analysis has an advantage because it avoids such problems. The study of Azizi et al. (2021) supported this point of view by comparing the source level and sensor level analysis methods in the classification problem of distinguishing patients with schizophrenia from healthy controls. The results showed that the best classifier performance was based on connectivity measures derived from the source space. Although it is possible to make a reasonable estimate of the source using sensor-level EEG localization, this approach is based on some physiological assumptions and has the natural limitations of single-mode imaging data. fMRI is an indirect measure of neural activity and is temporally limited by the slow hemodynamic response, thus it is unable to directly address neuronal activity within the cortex (Nguyen et al., 2019). Although EEG and fMRI single-modal studies have provided important insights into brain activity associated with emotional processing, their limitations have hampered the analysis of functional connectivity between regions involved in cognitive reappraisal.

To date, only a few studies provided mechanistic insights into emotion regulation by using simultaneous EEG-fMRI and even fewer investigated the EEG source-level connectivity with EEG-fMRI integration. First, simultaneous EEG-fMRI provides the ability to design novel emotion regulation neurofeedback paradigms, in which one modal can be used to extract the feedback neural index and the other modal can be used to conduct the brain function analysis, as well as to probe the activity changes after intervention. For example, in an EEG neurofeedback study, the effect of training in emotion regulation by retrieving positive autobiographical memories was evidenced by increased connectivity between the prefrontal, parietal, limbic, temporal, and occipital regions, showing more synchronized brain networks during neurofeedback (Dehghani et al., 2020a). This effect was validated by a study examining the fMRI brain connectivity and activity changes with the same experimental design, and increased activity in the prefrontal, occipital, parietal, and limbic regions was found (Dehghani et al., 2020b). The emotional regulation training can also be designed with real-time fMRI neurofeedback and concurrent EEG recordings. In an fMRI neurofeedback study, participants were instructed to upregulate left amygdala activity during happy memory recall, frontal EEG asymmetry was found to be correlated with left amygdala activity, and left fronto-temporal EEG coherence was found to be positively associated with decreased depression symptoms (Bodurka, 2018). In addition, the integration of EEG and fMRI can achieve in-depth information about the emotion regulation process due to their complementary characteristics of high spatial and temporal brain imaging (Rosa et al., 2010). In an EEG-informed fMRI study, the general linear modal (GLM) was used to investigate the cortical areas that modulate the frontal LPP when downregulating the negative affect by cognitive reappraisal. It was found that the septum pellucidum, the right insula, and the right subcallosal gyrus were involved in the modulation of the LPP amplitude (Fang et al., 2019). The integration of EEG and fMRI can also improve the localization of cortical sources with higher efficiency (Lin et al., 2004). When it comes to source-level functional connectivity analysis, combining EEG and fMRI can provide improved network characterization (Labounek et al., 2019). However, this kind of source-level brain network analysis method is seldom reported in cognitive reappraisal, except for a study by Nguyen et al. (2019) that examined the causal brain network associated with the emotion process, in which the ventrolateral prefrontal cortex (VLPFC) was found to play a modulator role in emotion network. Further studies are needed to reveal the detailed modulation effect on neural activity and network characteristics during reappraisal.

Source localization with scalp electroencephalography recordings is an inverse problem that tries to specify the location of the sources of the brain activity. There are mainly two kinds of methods to solve the inverse problem, namely, the parameterized method of the equivalent current dipole model (Tenney et al., 2020) and the non-parameterized method of distributed source model (Michel and He, 2019). Minimum norm estimate (MNE) is the classical method of the distributed source model (Xu et al., 2018). The statistical maps derived from fMRI data can be used as a spatial prior for the distributed source reconstruction, so the fMRI constrained EEG source imaging has attracted more and more attention. The fMRI-weighted minimum norm estimation (fMNE) algorithm is the mostly used method, in which fMRI spatial information is integrated into the EEG source location framework as *a priori* information (Xu et al., 2018). In our previous research, the fused priori weighted minimum norm estimation (FWMNE) was developed based on EEG sliding windows in order to effectively trace the brain source dynamics (Zhang et al., 2021).

In this study, we used the FWMNE method for the source localization by EEG-fMRI integration and estimated the dynamic cortical activity during cognitive reappraisal, which was then used to construct the emotional regulation network that supports the system interaction analysis. Our aim was to apply the proposed source localization method to the analysis of emotional processing and to investigate how the neural activity and brain network parameters were modulated by cognitive reappraisal. We hypothesized that the regions responsible for the cognitive processing of reappraisal would be activated, and the system interaction and operation of regulation would be reflected in network characteristics by graph theory.

## Experimental materials and methods

### Participants

A total of 20 subjects from Changzhou University were recruited, and 5 subjects were excluded from further analysis due to head movements and resulting low-quality EEG recordings. For the final 15 subjects included in this study, 12 were men and 3 were women. The age range of the participants was 19–24 years, with a mean of 21.8 and a standard deviation of 1.4 years. All participants had a normal or corrected-to-normal vision and no history of neurological or mental illness. The experimental protocol was approved by the research ethics committee of Changzhou University, and after explaining the considerations in the experiment, we obtained a signed written consent form from each participant according to the Declaration of Helsinki.

### Experimental paradigm

**Figure 1:**
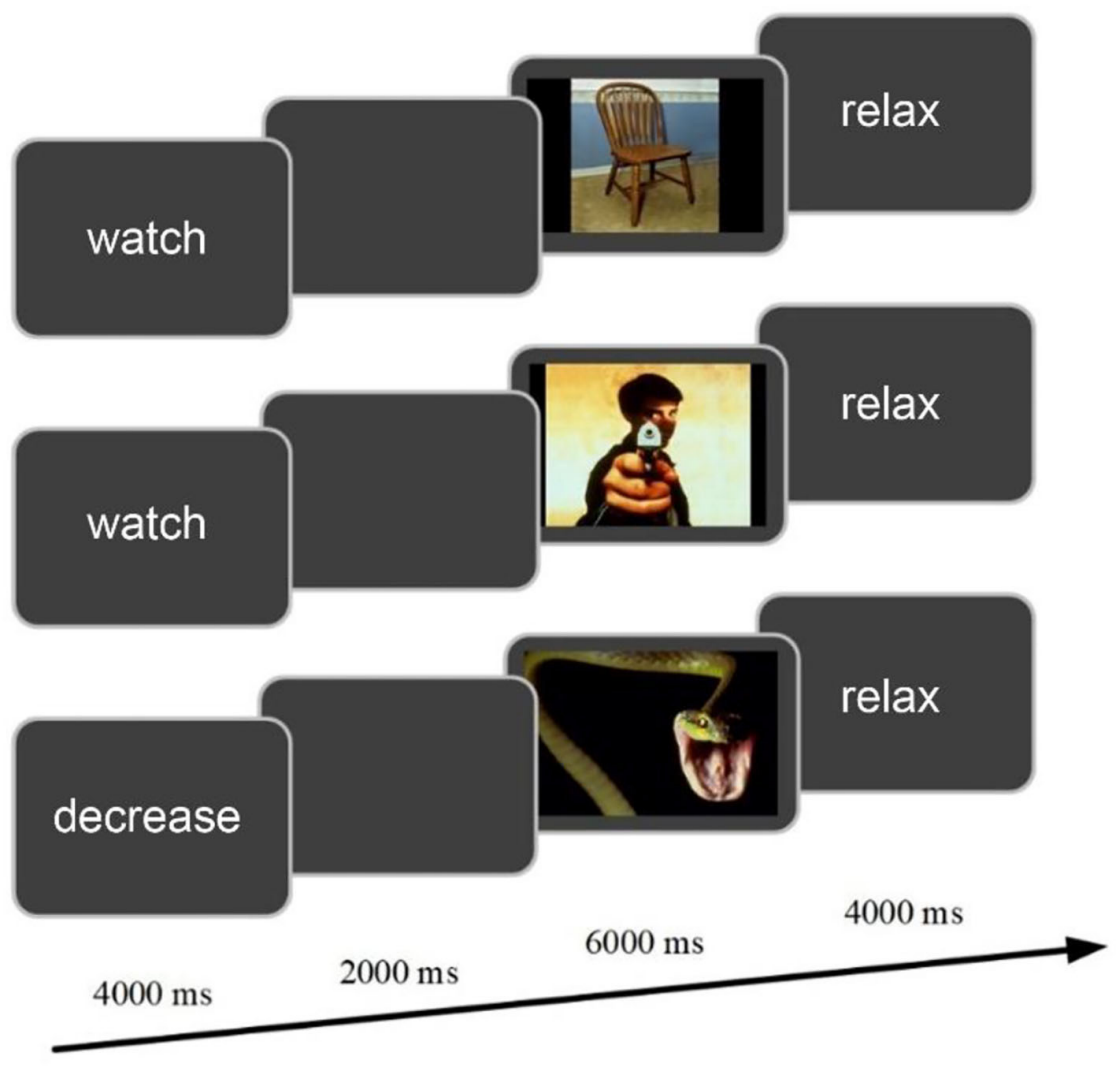
The experimental design of cognitive reappraisal task for a single trial.

The experiment involved emotional cognitive reappraisal task. As seen in Figure 1, the single experimental trial began with an instruction (either watch or decrease) in the center of the gray screen for 4 s, followed by a blank screen for 2 s. A color image was then displayed against the gray background for 6 s. After the offset of each image, the cue word “relax” was presented for 4 s, prompting participants to take rest. Visual stimuli were 120 emotional images selected from the International Affective Picture System (IAPS) (Lang et al., 1997). Among them, 40 images were neutral and used for the watch task (neutral-watch); 80 images were negative, equally divided into two parts, i.e., one part was for the watch task (negative-watch) and the other part was for the reappraisal task (negative-reappraisal). Participants should react naturally to the image in the watch task and try to reduce the degree of negative emotion in the decrease task by using the regulation strategy of reappraisal. The experimenters interpreted the concept of reappraisal with an example trial and described how it was possible to come up with a less negative interpretation of the image content (e.g., imagining the situation being unreal or assuming a better outcome of the scene). The participants were then trained with several trials, which were not used in the formal task. The participants reported their reinterpretations to the experimenter, and the experimenter determined whether the participants correctly reappraised the negative images according to the experimental criteria. In the formal task, a total of 120 trials were equally divided into four sessions. Each session included 10 neutral-watch, 10 negative-watch, and 10 negative-reappraisal trials. The sequence of the 30 trials in each session was randomized for each subject.

### Simultaneous EEG-fMRI data recording

The experiment with synchronous EEG-fMRI was conducted in Changzhou Second People's Hospital. Participants were scanned with a 3-T scanner (Philips Medical Systems) while wearing an EEG-Cap (HydroCel Geodesic Sensor Net; Electrical Geodesics, Inc., Eugene, OR). EEG data were collected by using Net Station software from 64 channels in 10-10 montages at the sampling rate of 250 Hz. Cz served as the reference. The impedance of all electrodes was kept below 50 kΩ. Functional MRI data acquisition (3-T scanner, Philips Medical System) was performed using a gradient echo EchoPlanar Imaging sequence [repetition time (TR) = 2,000 ms, echo time (TE) = 35 ms, flip angle = 90°, and voxel size = 3 × 3 × 3 mm]. A total of 24 continuous slices parallel to the anterior commissure-posterior commissure line were acquired per volume (field of view of 230 × 182 mm and matrix of 96 × 74). A structural MRI image was also collected from each participant with a voxel size of 1 × 1 × 1 mm. The EEG and fMRI data were synchronized using a synchronization box.

### Pre-processing

EEG data were processed by using the Netstation software. First, the average artifact subtraction (AAS) algorithm (Allen et al., 2000) was used to perform gradient artifact correction, and the optimal basis set (OBS) algorithm (Niazy et al., 2005) was applied to suppress ballistocardiogram artifacts. Next, the FIR filter with the passband of 0.01–40 Hz was applied. Then, the EEG data were re-referenced to the common grand average of all EEG channels, segmented to −0.2 to 1.5 s epochs relative to the image onset, and baseline corrected against the −200 ms to 0 ms. Furthermore, artifact detection and bad channel replacement were applied to each channel and segment. Finally, an independent component analysis (ICA) algorithm was adopted to remove residual noises, such as electromyography, eye movement, and head movement artifacts. The ADJUST plug-in (automatic EEG artifact detector based on the joint use of spatial and temporal features) was adopted to help remove the residual noise component (Mognon et al., 2011).

The fMRI data were preprocessed and analyzed with the SPM8 software (http://www.fil.ion.ucl.ac.uk/spm/, RRID:SCR_007037). The preprocessing steps include slice time correction, realignment, head motion correction, spatial normalization, and spatial smoothing. First, the fMRI images were corrected for slice-timing artifacts and spatially realigned to the first brain volume. To exclude the head motion effects, subjects showing a maximum displacement of > 2 mm and an angular motion of >2° were removed. Under this criterion, five subjects were excluded. Then, the data were normalized based on the Montreal Neurologic Institute (MNI) reference brain, and the voxel sizes were turned into 3 × 3 × 3 mm. In addition, fMRI maps were smoothed by an 8-mm FWHM Gaussian kernel. Finally, the images were filtered with a temporal band-pass of 0.01–0.08 Hz.

### Analytical framework of the cognitive reappraisal network

**Figure 2:**
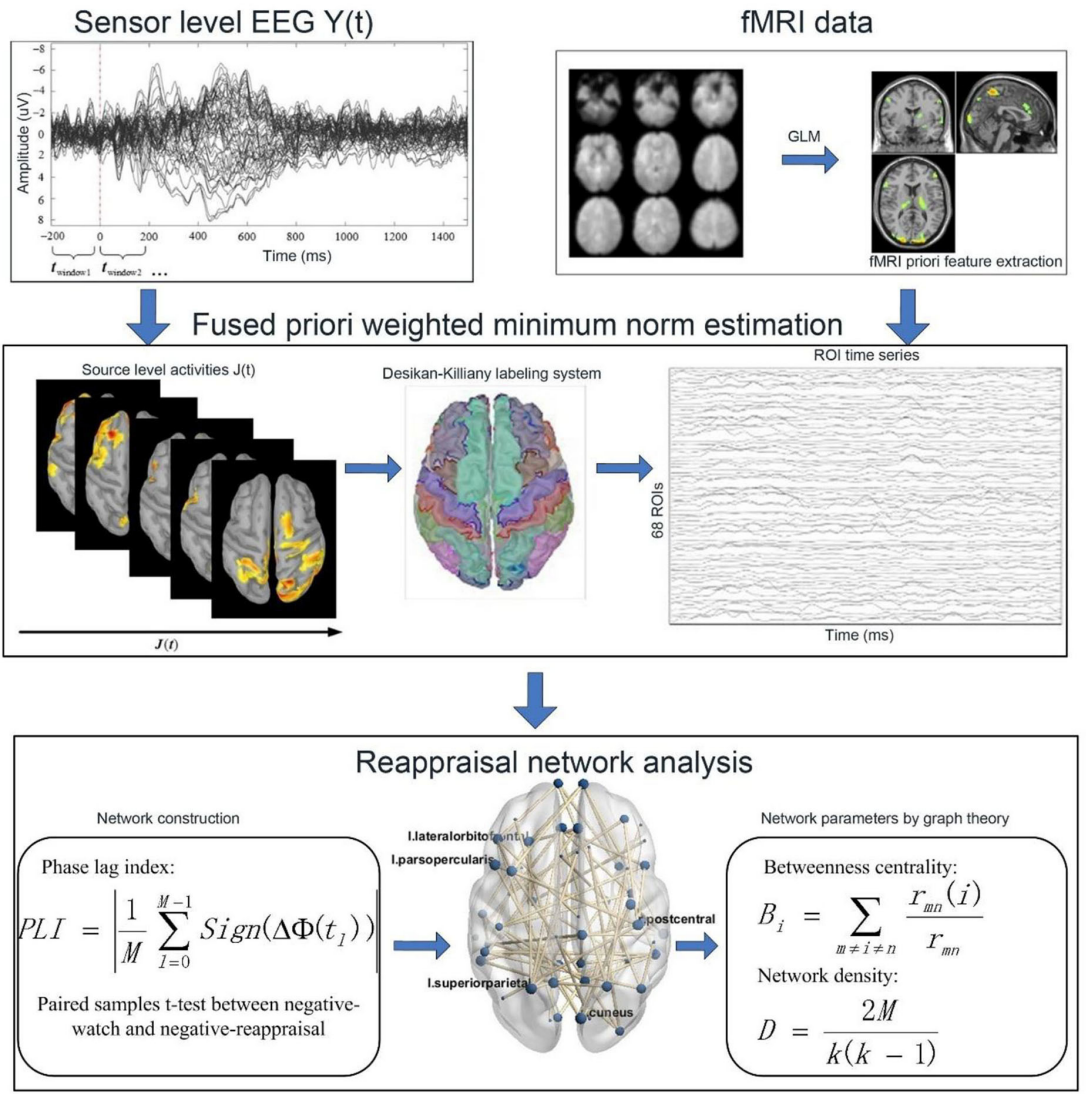
Flowchart of the analytical framework.

The flowchart of the framework used in the reappraisal network analysis is shown in Figure 2. First, we used the FWMNE method proposed in our previous research (Zhang et al., 2021) to project sensor-level EEG to source space. In the fMRI priori features extraction, the predicted BOLD signals were recorded by convolving the extracted EEG features with standard hemodynamic response function (HRF) and used as EEG regressors. The GLM was used in the EEG-informed fMRI analysis to find the task-relevant activations in the whole brain, which were taken as fMRI priori features (Zhang et al., 2021). Next, the Desikan-Killiany labeling system (Desikan et al., 2006) was used to extract the ROI time series from source space activities. Then, the phase lag index (PLI) (Stam et al., 2007) was used to measure the synchronization between ROIs. A paired sample *t*-test was used to identify the connectivity with a significant difference between negative-watch and negative-reappraisal conditions (*df* = 14; FDR-corrected for multiple comparisons; *q* = 0.05), which formed the edges of the network. Finally, the reappraisal network parameters were analyzed using graph theory.

### Source localization and analysis

The classical EEG inverse problem is depicted as follows (Nguyen et al., 2018):


$$\begin{array}{l} \begin{array}{l} \;Y=GJ+\varepsilon \\ \varepsilon \sim N(0,C,T)\\ J\sim N(0,R,T), \end{array} \end{array}$$

where *G* ∈ R^*m*×*s*^ is the lead field matrix, and *J* ∈ R^*s*×*d*^ is the unknown brain activities in the source space with *s* dipoles. ε represents the noise component in the sensor space, and *C* ∈ R^*m*×*m*^ represents the noise spatial covariance matrix. *R* ∈ R^*s*×*s*^ is the source space covariance matrix of *J*, representing prior knowledge about the distribution. The source current vector *J* is estimated as follows:


$$\begin{array}{l} \hat{J}=RG^{T}{\left(GRG^{T}+C\right)}^{-1}Y \end{array}$$

In fMRI-weighted minimum norm estimation, prior information can be extracted from fMRI statistical analysis, and the dipole covariance matrix can be set as follows:


$$\begin{array}{l} R=R_{f} \end{array}$$

In *R*_*f*_, the diagonal elements are set to 1 for the locations in the activation regions, and other diagonal elements are set to 0.1, while the off-diagonal elements are set to 0 (Xu et al., 2018).

After obtaining *J*_*fMNE*_ by using the fMNE method, we divided EEG with sliding time windows and extracted the fusion feature of the dipole in each time window, functioning as dynamic fused prior information for weighted minimum norm estimation. The new covariance matrix *R*_*window*_ was constructed with the diagonal elements set as:


$$\begin{array}{l} \begin{array}{l} {\hat{R}}_{window}=diag(cov(J_{fMNE\_window}^{T})) \end{array}, \end{array}$$

where *J*_*fMNE*_*window*_ is the source space activities in each time window using the fMNE method. After that, the fused priori weighted minimum norm estimations were performed, and the source activity in each time window was estimated as follows:


$$\begin{array}{l} J_{window}={R_{window}G^{T}\left(GR_{window}G^{T}+C\right)}^{-1}Y_{window} \end{array}$$

The analysis was performed in each of the three windows: 60–172 ms, 428–580 ms, and 1,164–1,276 ms, because the statistical analysis of the parietal-occipital event-related potential showed that the amplitude was lower in negative-reappraisal than in the negative-watch condition in the three windows (Zhang et al., 2021).

### Phase lag index

We used the phase lag index to measure the synchronicity in the neural activity between all possible pairs of source-level regions of interest (ROIs), and it is defined as follows (Stam et al., 2007):


$$\begin{array}{l} PLI=|\frac{1}{M}\overset{M-1}{\underset{l=0}{\sum }}Sign\left(\text{Δ}\emptyset \left(t_{l}\;\right)\right)|, \end{array}$$

where M is the time series length, and Δ∅(*t*_*l*_), *l* = 0, 1, …, *M–1* represent a time series of phase differences, which is defined as follows:

Δ∅(*t*_*l*_) = ∅_*j*_(*t*_*l*_)−∅_*k*_(*t*_*l*_), where ∅_*j*_ and ∅_*k*_ are the instantaneous phases of time series *j* and *k*, and extracted using the analytical signal:


$$\begin{array}{l} x^{H}\left(t\right)=x\left(t\right)+i\tilde{x}\left(t\right) \end{array}$$

*x*(*t*) is the original time series, and $\tilde{x}\left(t\right)$ is its Hilbert transform. The instantaneous phase can be calculated as follows:


$$\begin{array}{l} \emptyset \left(t\right)=\arctan\frac{\tilde{x}\left(t\right)}{x\left(t\right)} \end{array}$$

The PLI is a bounded measure between 0 and 1, where 0 represented no phase synchronization and 1 indicated a fixed phase relationship.

In the current study, the PLI values were calculated between any two ROI time series in five frequency bands, namely, delta (1–3 Hz), theta (3–8 Hz), alpha (8–13 Hz), beta (13–30 Hz), and gamma (30–40 Hz), and averaged across trials for each condition in each participant (Yin et al., 2020), based on which the PLI matrix was constructed.

### Brain network analysis

#### Brain network construction

According to the Desikan-Killiany labeling system (Desikan et al., 2006), we extracted 68 ROIs from brain activities in the source space, which were defined as nodes in the network. The PLI method was used to construct the connectivity matrix, which was later converted to a binary graph representation of the brain network by considering a threshold. The threshold was determined according to the following criteria: after binarization, the average degree of nodes is > 2*lnK* = 2ln(68) ≈ 8.4, where *k* represents the number of network nodes, and the small-world index is >1 (Zhang et al., 2011). Finally, the threshold was set as 0.30. A paired sample *t*-test was performed on each connectivity between negative-reappraisal and negative-watch conditions, and the edges with a significant difference (*df* = 14; FDR-corrected for multiple comparison; *q* = 0.05) were retained for network measures analysis.

#### Betweenness centrality

Betweenness centrality quantifies the number of times a node acts as a bridge over the shortest path between two other nodes. It can also be considered as a measure for quantifying the importance of one node to the communication between two other nodes. It measures the global characteristics of nodes in the network and reflects the degree to which a node (brain region) acts as an efficient relay within the network (Fang et al., 2020). The betweenness centrality of node *i* can be calculated as follows:

$B_{i}=\underset{m\neq i\neq n}{\sum }\frac{r_{mn}\left(i\right)}{r_{mn}}$, where *r*_*mn*_ is the total number of shortest paths from node *m* to node *n*, and *r*_*mn*_(*i*) is the number of paths that go through node *i*.

#### Network density

Network density is a measure quantifying the density of a functional brain network. It can be calculated as follows:

$D=\frac{2M}{K\left(K-1\right)}$, where K represents the number of nodes, and M represents the number of actual edges.

## Results

**Figure 3:**
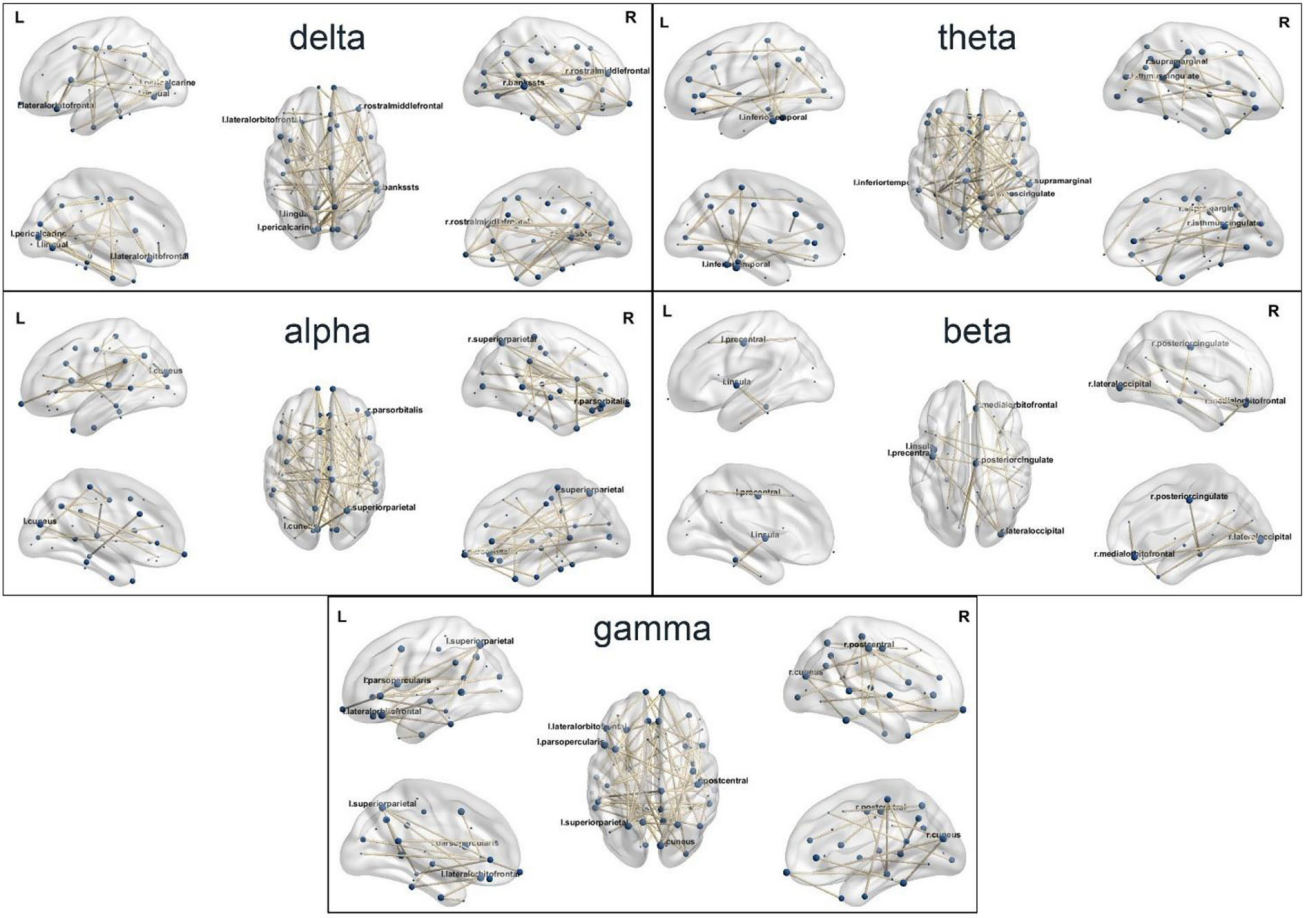
The source-level brain networks of cognitive reappraisal in five frequency bands.

The brain networks in delta, theta, alpha, beta, and gamma bands are demonstrated in Figure 3. The important nodes, measured by betweenness centrality, are illustrated with blue dots in the network (Figure 3 and Table 1). In the delta band, the banks of the superior temporal sulcus (right), the pericalcarine (left), the lingual gyrus (left), the lateral orbitofrontal cortex (left), and the rostral middle frontal gyrus (right) were the brain regions with high betweenness centrality, suggesting that these regions played an important role in information transmission during cognitive reappraisal. In the theta band, the regions with high betweenness centrality included the isthmus of the cingulate gyrus (right), the inferior temporal cortex (left), and the supramarginal gyrus (right). In the alpha band, the superior parietal gyrus (right), the cuneus (left), and the pars orbitalis (right) were the central nodes of the network. The betweenness centrality in the beta band was relatively lower compared with other bands, and the important regions included the lateral occipital sulcus (right), the posterior cingulate cortex (right), the precentral gyrus (left), and the medial orbitofrontal cortex (right). In the gamma band, the emotional cognitive reappraisal network, the cuneus (right), the lateral orbitofrontal cortex (left), the superior parietal gyrus (left), the postcentral gyrus (right), and the pars opercularis (left) were the brain regions with large betweenness centrality, representing the central nodes of the network. The network density in delta, theta, alpha, and gamma bands was similar, while the network density in the beta band was relatively low.

**Table 1:** Brain regions with high betweenness centrality in the cognitive reappraisal networks.

**Frequency bands**	**Region**	**Desikan-killiany label**	**Betweenness centrality**
Delta	Banks of the superior temporal sulcus (right)	r.bankssts	939.979
	Pericalcarine (left)	l.pericalcarine	608.266
	Lingual gyrus (left)	l.lingual	406.736
	Lateral orbitofrontal cortex (left)	l.lateralorbitofrontal	333.695
	Rostral middle frontal gyrus (right)	r.rostralmiddlefrontal	280.742
Theta	Isthmus of cingulate gyrus (right)	r.isthmuscingulate	990.901
	Inferior temporal cortex (left)	l.inferiortemporal	775.866
	Supramarginal gyrus (right)	r.supramarginal	613.933
Alpha	Superior parietal gyrus (right)	r.superiorparietal	1227.661
	Cuneus (left)	l.cuneus	961.647
	Pars orbitalis (right)	r.parsorbitalis	682.633
Beta	Lateral occipital sulcus (right)	r.lateraloccipital	82.102
	Posterior cingulate cortex (right)	r.posteriorcingulate	74.524
	Precentral gyrus (left)	l.precentral	70.613
	Medial orbitofrontal cortex (right)	r.medialorbitofrontal	64.877
Gamma	Cuneus (right)	r.cuneus	816.549
	Lateral orbitofrontal cortex (left)	l.lateralorbitofrontal	760.86
	Superior parietal gyrus (left)	l.superiorparietal	635.769
	Postcentral gyrus (right)	r.postcentral	549.215
	Pars opercularis (left)	l.parsopercularis	548.215

## Discussion

According to Gross's process model, emotion may be regulated at five points in the emotion generative process: selection of the situation, modification of the situation, deployment of attention, change of cognitions, and modulation of experiential, behavioral, or physiological responses (Gross, 2002). In cognitive reappraisal, the stimulus is perceived in the context of the current situation at first. Then, people attend to some of these stimuli or their attributes. The next step involves assessing the importance and relevance of the stimulus. Finally, these appraisals were translated into a change in emotional experience (Ochsner et al., 2012). A number of brain regions take part in the cognitive reappraisal process, including the inferior parietal regions and the dorsolateral and posterior prefrontal cortices that are associated with selective attention and working memory, the dorsal anterior cingulate cortex involved in performance monitoring, regions of the ventrolateral prefrontal cortex involved in appropriate target selection and inappropriate target suppression responses, and the dorsomedial prefrontal regions implicated in attributing mental states (Ochsner et al., 2012).

In our previous study by Zhang et al. (2021), the activation of the specific brain regions in cognitive reappraisal and its dynamic changes were examined by conducting the contrast between negative-reappraisal and negative-watch in three important time windows. Neural modulation can be detected from the activation change when the affective response of the negative images was regulated. In 60–172 ms, the left inferior parietal lobe demonstrated higher significant activations during cognitive reappraisal, which was in line with previous studies. In the fMRI study by Lee et al. (2021), it was found that cognitive reappraisal induced higher activities in the bilateral inferior parietal lobes compared to the condition of “look.” The inferior parietal lobe was also identified in the downregulation of negative emotion compared with just looking (Ochsner et al., 2004). Modulation of inferior parietal cortices may reflect the attentional selection in working memory. In 428–580 ms, the right superior temporal gyrus demonstrated higher significant activations in reappraisal, consistent with the study by Ochsner et al. (2004) comparing the activity between reappraisal and “look”. It has been indicated that the superior temporal gyrus should be involved in the execution of regulation initiated by the frontal areas (Kohn et al., 2014). So, we inferred that the cortical activation of the right superior temporal gyrus indicated the execution of the reappraisal. Besides, we also found that reappraisal was associated with the modulation of the lateral occipital cortex, reflecting the role of perceptual processing (Lake et al., 2017). In 1,164–1,276 ms, it was found that reappraisal processing induced higher activation in the right inferior middle frontal gyrus, an area associated with cognitive reappraisal during emotion regulation (Wadden et al., 2018). While in contrast to negative-watch > negative reappraisal, it was found that activation of the insula was significantly lower during reappraisal than during just looking. Ochsner et al. (2004) found modulated activity in the bilateral insular cortex when decreasing the negative affect, showing decreased activity in reappraisal. We inferred that this modulation indicated a decreased negative affect by reappraisal.

The process for the emotional processing and regulation of negative affect involves the interactions between several cognitive processes in the neural system, resulting in the recruitment of a large-scale functional brain network (Schlumpf et al., 2019; Fang et al., 2020). In this study, based on our previous source localization results (Zhang et al., 2021), functional networks were constructed at the source level in different frequency bands. The central nodes of the emotional reappraisal network were identified by using the graph theory measure of betweenness centrality, and the density of the brain network in different frequency bands was analyzed. It has been indicated that the gamma band plays an important role in the cognitive control of emotions (Oathes et al., 2008; Yang et al., 2020). In this study, the left lateral orbitofrontal cortex, the left superior parietal gyrus, the right cuneus, the right postcentral gyrus, and the left pars opercularis were identified as central nodes in the gamma band reappraisal network. In the study by Gao et al. (2021), the functional coupling of the orbitofrontal cortex and the basolateral amygdala was found to mediate the association between spontaneous reappraisal and emotional response. The left superior parietal gyrus is a part of attention and executive control networks, coordinating attention under competing conditions and voluntary orienting of attention (Corbetta and Shulman, 2002). The functioning of the left superior parietal gyrus seemed to be associated with cognitive reappraisal during emotion regulation. In the study by Wadden et al. (2018), yoga practitioners uniquely activated clusters of voxels in the left superior parietal lobule during emotion regulation. Reappraisal also generated significant responses in cuneus. In the study by Goldin et al. (2008), higher activity was found in bilateral cuneus in reappraisal compared with suppress strategy. These findings may give light to the study on brain networks related to emotion regulation.

## Conclusion

In this investigation, the aim was to examine the source-level functional network of emotional cognitive reappraisal by using the fused priori weighted minimum norm estimation method and the phase lag index. We constructed the network at different frequency bands. The gamma band network was found to be closely related to reappraisal. The central nodes were identified by the betweenness centrality, including the left lateral orbitofrontal cortex, the left superior parietal gyrus, the right cuneus, and so on. The source-level connectivity analysis method proposed in this study was applicable in examining the neural activity of the cognitive process, and the analysis of emotion regulation networks would give light to the research on mechanisms of emotional cognitive reappraisal.

## Data availability statement

The original contributions presented in the study are included in the article/supplementary material, further inquiries can be directed to the corresponding author.

## Ethics statement

The studies involving human participants were reviewed and approved by the research Ethics Committee of Changzhou University. The patients/participants provided their written informed consent to participate in this study.

## Author contributions

WL: methodology, software, validation, formal analysis, and writing—original draft. WZ: investigation, data curation, methodology, and visualization. ZJ: methodology. TZ: software. SX: resources. LZ: conceptualization, resources, writing—review and editing, supervision, project administration, and funding acquisition. All authors contributed to the article and approved the submitted version.

## Funding

This work is partly supported by the project of Jiangsu Key Research and Development Plan (BE2021012-2 and BE2021012-5), Key Laboratory of Brain Machine Collaborative Intelligence Foundation of Zhejiang Province (2020E10010-04), and Human-Machine Intelligence and Interaction International Joint Laboratory Project and Changzhou Sci&Tech Program (Grant Nos. CE20215026 and CE20225034).

## Conflict of interest

The authors declare that the research was conducted in the absence of any commercial or financial relationships that could be construed as a potential conflict of interest.

## Publisher's note

All claims expressed in this article are solely those of the authors and do not necessarily represent those of their affiliated organizations, or those of the publisher, the editors and the reviewers. Any product that may be evaluated in this article, or claim that may be made by its manufacturer, is not guaranteed or endorsed by the publisher.
